# Formation of Co–O bonds and reversal of thermal annealing effects induced by X-ray irradiation in (Y, Co)-codoped CeO_2_ nanocrystals

**DOI:** 10.1038/s41598-022-05691-0

**Published:** 2022-01-28

**Authors:** Tai-Sing Wu, Sheng-Fu Chen, Shih-Chang Weng, Yun-Liang Soo

**Affiliations:** 1 National Synchrotron Radiation Research Center, Hsinchu, Taiwan; 2 Department of Physics, National Tsing Hua University, Hsinchu, Taiwan

**Keywords:** Chemistry, Catalysis, Photocatalysis, Structural properties, Materials science, Condensed-matter physics, Nanoscale materials, Structural materials, Chemical physics, Physics, Condensed-matter physics, Structure of solids and liquids

## Abstract

We report an unconventional effect of synchrotron X-ray irradiation in which Co–O bonds in thermally annealed (Y, Co)-codoped CeO_2_ nanocrystal samples were formed due to, instead of broken by, X-ray irradiation. Our experimental data indicate that escaping oxygen atoms from X-ray-broken Ce–O bonds may be captured by Co dopant atoms to form additional Co–O bonds. Consequently, the Co dopant atoms were *pumped* by X-rays from the energetically-favored thermally-stable Co-O4 square-planar structure to the metastable octahedral Co-O6 environment, practically a reversal of thermal annealing effects in (Y, Co)-codoped CeO_2_ nanocrystals. The band gap of doped CeO_2_ with Co dopant in the Co-O6 structure was previously found to be 1.61 eV higher than that with Co in the Co-O4 environment. Therefore, X-ray irradiation can work with thermal annealing in opposing directions to fine tune and optimize the band gap of the material for specific technological applications.

## Introduction

It is well known that X-rays can break chemical bonds and therefore cause radiation damage in crystalline materials. For example, damage to disulfide bonds and iron–water bonds due to X-rays was found in synchrotron protein crystallography measurements^1–3^. Similar chemical-bond-breaking effects of synchrotron X-rays has also been reported for small molecules^4^. By contrast, inorganic materials are considered to be more stable under X-ray irradiation. Nevertheless, phase transitions due to radiation damages may also occur in inorganic materials under intense X-ray irradiation^5–8^.

The CeO_2_-based nanocrystals has been widely used as photocatalysts in many energy and environment related applications in which the band gap of the catalyst plays a crucially important role^9–12^. We have previously demonstrated that shinning synchrotron X-rays on CeO_2_ nanocrystals can break Ce–O bonds in large numbers, an effect that can be further enhanced by doping subvalent Y dopant atoms into the material to generate oxygen vacancy pathways for more efficient escape of the breakaway oxygen atoms from the broken Ce–O bonds^13^. We have also shown that Co dopant atoms have two distinct types of oxygen coordination in the CeO_2_ nanocrystalline host, namely the square-planar coordination and the octahedral coordination, in which cobalt atoms are bonded to 4 and 6 oxygen nearest neighboring atoms, respectively. We have observed that thermal annealing at elevated temperatures can break Co–O bonds, leading to rearrangement of oxygen coordination around Co dopant atoms from the metastable octahedral coordination to the energetically favored square-planar coordination^14^.

The interplay between the bond-breaking effect of X-rays on the Ce–O bonds and that of thermal annealing on the Co–O bonds assisted by Y-codoping described above appears to be an intriguing issue for modulating the physical properties of the Co-doped CeO_2_ system for technological applications. In this paper, we observe the effect of X-ray irradiation on the Co–O bonds in (Y, Co)-codoped CeO_2_ nanocrystal samples where the breaking of Co–O bonds due to thermal annealing has reduced the oxygen coordination number around Co from 6 to 4. We present an unprecedented effect of X-ray irradiation in which Co–O bonds were formed due to, instead of broken by, X-ray irradiation. The predominantly square planar (Co-O4) oxygen coordination surrounding cobalt atoms in thermally annealed (Y, Co)-codoped CeO_2_ nanocrystals was largely switched back to the octahedral (Co-O6) coordination after prolonged X-ray irradiation. Therefore, thermal annealing and X-ray irradiation can act in opposing directions to adjust the compositions of the square-planar and octahedral types of Co oxygen coordination to optimize the properties of the material for specific applications. As demonstrated in our previous works, introduction of Co dopant atoms can switch-on substantial decrease of band gap with increasing Y codopant concentration^15^ and rearrangement of oxygen coordination around Co dopant atoms can lead to a dramatic band gap variation of 1.61 eV^14^ in the CeO_2_ host. We have thus developed a unique method to fine tune the band-gap-related electronic and optical properties of (Y, Co)-codoped CeO_2_ nanocrystals using thermal annealing and X-ray irradiation for various technological applications.

## Experimental

**Figure 1:**
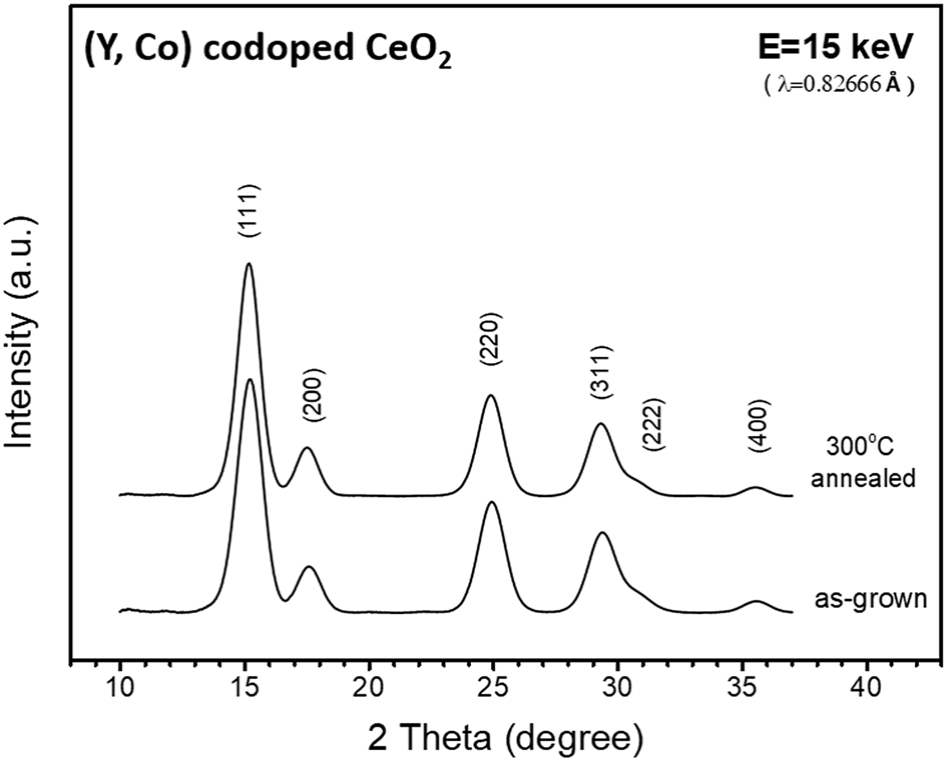
XRD patterns of the as-grown and thermally annealed samples. The X-ray photon energy selected for these measurements is 15 keV.

Samples of (Y, Co)-codoped CeO_2_ nanocrystals were synthesized using a polyol method^15^. The as-grown samples were then annealed under pure O_2_ at 300 °C for 30 min with a ramping rate of 10 °C/min such that the square-planar oxygen coordination is dominant surrounding the Co dopant atoms^14^. The Y and Co concentrations of the sample are 36.9 and 0.6 at. %, respectively, as determined from inductively coupled plasma mass spectrometry (ICPMS) measurements. As shown in Fig. 1, the synchrotron-based X-ray powder diffraction (XRD) data indicate that both the as-grown and annealed samples have good crystallinity. Despite the heavy Y doping, the XRD patterns match well with that of cubic CeO_2_ at the (111), (200), (220), (311), (222) and (400) Bragg peaks. The crystallite sizes of the as-grown and annealed samples were determined from XRD using the Scherrer equation to be 3.7 and 3.9 nm, respectively. To investigate the effect of X-ray irradiation, the thermally annealed sample was irradiated with synchrotron X-ray beam at photon energy 7 keV. The spot size of the X-ray beam on the sample is 1.5 mm × 0.5 mm and the photon flux is 1.2 × 10^12^ photons per second. Local structures surrounding the cobalt dopant atoms in the as-grown, annealed, and X-ray irradiated samples were probed by using Co-K-edge X-ray absorption near edge structure (XANES) and extended X-ray absorption fine structure (EXAFS) techniques. For comparison, the Ce-L_3_-edge and Y-K-edge XAFS (XANES and EXAFS) data for the above samples were also measured and analyzed, shown as supplementary information online. Cerium L_3_-edge and Co K-edge XANES data of samples irradiated for different time durations were measured to elucidate the relation between the increase of oxygen vacancies surrounding the Ce constituent atoms of the host due to X-ray irradiation and the change of oxygen coordination surrounding the Co dopant atoms. All X-ray measurements and treatments were performed at beamline 07A of Taiwan Light Source (TLS) at National Synchrotron Radiation Research Center (NSRRC) in Taiwan.

## Results and discussion

**Figure 2:**
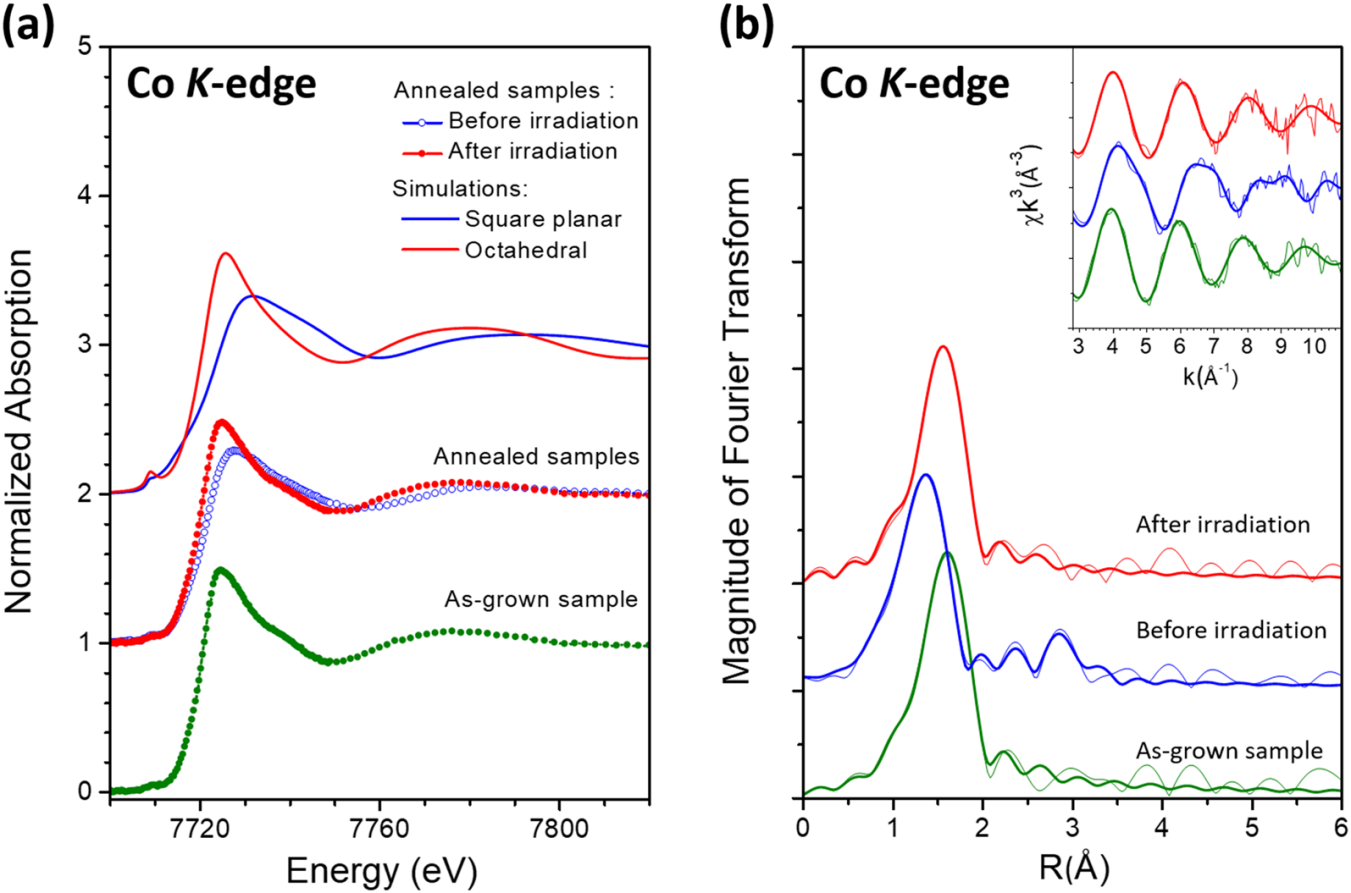
(**a**) Theoretical and experimental Co K-edge XANES curves for (Y, Co)-codoped CeO_2_ samples. (**b**) Co K-edge EXAFS data for (Y, Co)-codoped CeO_2_ samples. Fine lines: experimental; Coarse lines: curve fitting. Curves have been shifted vertically for the sake of clarity.

As shown in Fig. 2a, the XANES curve of the as-grown sample is very different from that of the sample annealed under O_2_ at 300 °C for 30 min. When the annealed sample was further treated with X-ray irradiation for 4 h, its XANES curve largely resumed the signatures of that of the as-grown sample. Since the Co K-edge XANES data basically represent the average local chemical environment surrounding the Co dopant atoms, we can see that the Co local structure in the as-grown sample was substantially altered by thermal annealing and then restored by the subsequent X-ray irradiation. As reported in one of our papers, the local structures of Co dopant atoms exhibit bistability in the CeO_2_ host. Thermal annealing was found to switch the local coordination environment from the initial octahedral structure (Co-O6) to the thermally stable square-planner (Co-O4) structure^14^. The XANES curves in Fig. 2a for the as-grown sample and the thermally annealed sample after X-ray irradiation resemble that theoretically simulated for the octahedral structure while that for the annealed sample before irradiation resembles that simulated for the square-planner structure. Detailed theoretical simulation using the FDMNES package is described in the supplementary information online^16,17^. Therefore, our XANES results indicate that X-ray irradiation can restore the octahedral oxygen coordination surrounding the Co atoms in the sample after it was switched to the square-planner coordination by thermal annealing the as-grown sample.

The Co K-edge EXAFS data analysis was performed using the *ARTEMIS* package^18^. The Fourier transforms (FT) of the k^3^-weighted EXAFS χ-functions from 2.8 to 10.5 Å^−1^ shown in Fig. 2b exhibit one pronounced peak for all three samples representing the nearest neighboring shells surrounding the Co dopant atoms. The EXAFS curve-fitting results listed in Table 1 demonstrate that the pronounced peak in the FT for the as-grown sample is due to 6.0 ± 0.3 O neighboring atoms at a distance 2.07 ± 0.01 Å from the central Co atom while that for the thermally annealed sample is a composite peak due to 4.0 ± 0.4 O and 1.4 ± 0.3 Ce atoms at distances 1.87 ± 0.01 Å and 3.21 ± 0.01 Å from the Co atom, respectively. The Co K-edge EXAFS results for the as-grown and thermally annealed sample are consistent with the octahedral and square-planner models of O coordination surrounding Co, respectively. After the annealed sample is treated with X-ray irradiation, the pronounced peak in the FT is ascribed to 5.4 ± 0.1 O atoms at a distance of 2.04 ± 0.01 Å indicating the O coordination around Co was largely switched from the square-planner structure of the annealed sample back to the octahedral structure of the as-grown sample.

**Table 1:** Parameters of local structure around Co atoms obtained from curve-fitting the Co K-edge EXAFS.

Sample	Bond	*N*	*R*	σ^2^	ΔE_0_	R-factor
			(Å)	(10^−3^ Å^2^)	(eV)	(%)
As-grown	Co–O	6.0 ± 0.3	2.07 ± 0.01	10.6 ± 0.7	1.8 ± 0.7	0.0012
Before X-ray	Co–O	4.0 ± 0.4	1.87 ± 0.01	13.1 ± 1.2	− 8.2 ± 1.6	0.0323
	Co–Ce	1.4 ± 0.3	3.21 ± 0.01	9.8 ± 1.9	− 7.0 ± 2.0	
After X-ray	Co–O	5.4 ± 0.1	2.04 ± 0.01	9.8 ± 0.3	0.0 ± 0.4	0.0007

N is the coordination number. R is the bond length. σ^2^ is the Debye–Waller-like factor serving as a measure of local disorder. ΔE_0_ is the difference between the zero kinetic energy value of the sample and that of the theoretical model used in FEFF. R-factor is a residual factor representing the goodness of fit. Uncertainties were estimated by the double-minimum residue (2χ^2^) method. The amplitude reduction factor ($S_{0}^{2}$) representing the central atom shakeup and shakeoff effects used in the curve-fitting is 0.72 as obtained from our previous papers^14^.

**Figure 3:**
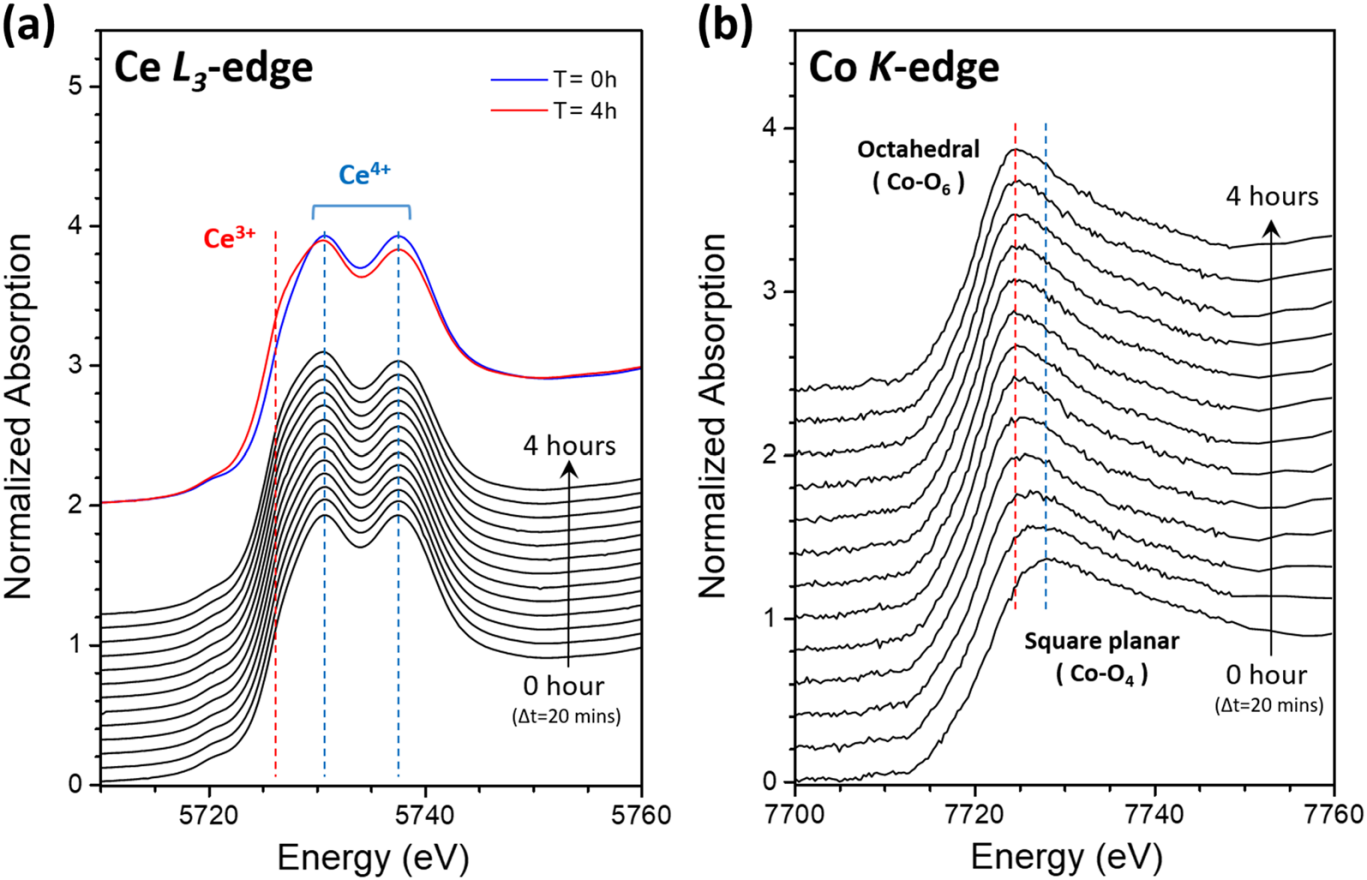
(**a**) Ce L_3_-edge and (**b**) Co K-edge XANES data for annealed (Y, Co) codoped doped CeO_2_ sample with different irradiation time. Δ*t* is the increment of irradiation time.

**Figure 4:**
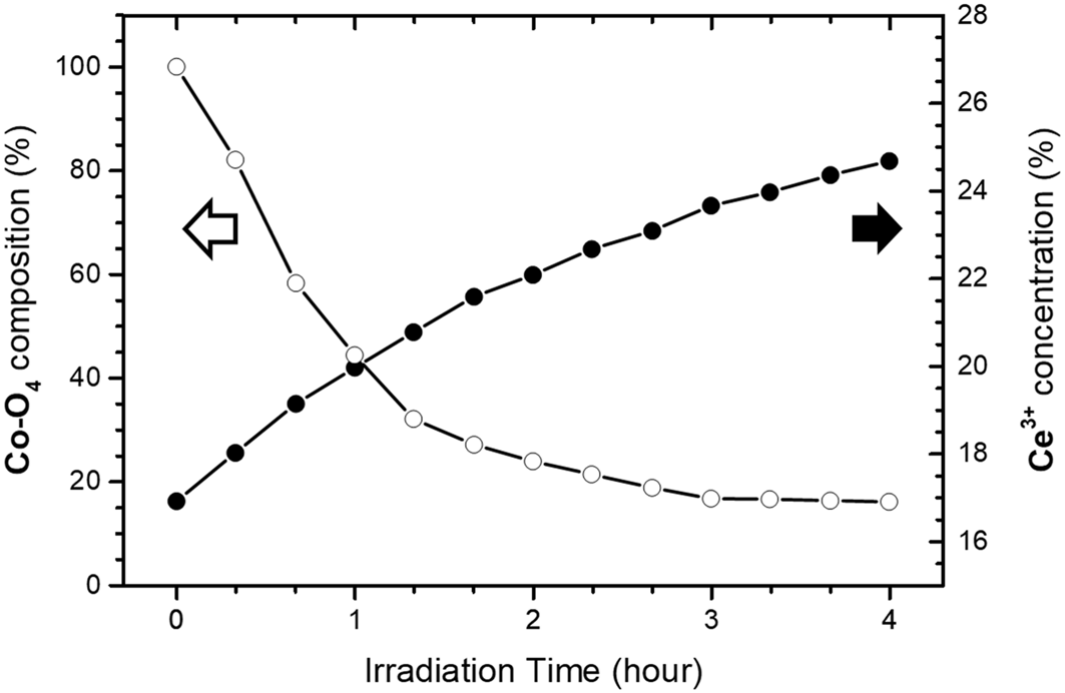
Plot of Ce^3+^ concentration and Co-O4 composition vs. irradiation time.

The Ce L_3_-edge and Co K-edge XANES curves of samples irradiated for different time durations are plotted in Fig. 3a and b, respectively. Curve-fittings using an arctangent function to simulate the edge jump and Gaussian functions for peak features were carried out to extract Ce^3+^ concentration from the Ce L_3_-edge XANES spectra. Details of the curve-fitting method have been reported in one of our previous papers^19^. The Co-O4 composition for each irradiated sample was estimated by curve-fitting its respective Co XANES spectrum as a linear combination of the XANES spectra for the as-grown and thermally annealed samples as the model curves for the Co-O6 and Co-O4 structures, respectively. As shown in Fig. 4, the Ce^3+^ concentration increases with irradiation time indicating that O atoms are breaking away from Ce atoms as the (Y,Co)-codoped CeO_2_ sample is being irradiated with X-rays. On the other hand, the Co-O4 composition of the sample decreases with irradiation time indicating the square-planar O coordination surrounding Co (Co-O4) atoms in the thermally annealed sample is being switched back to the octahedral O coordination (Co-O6) as a result of X-ray irradiation.

The Ce L_3_-edge and Y K-edge XAFS (XANES and EXAFS) data for the as-grown, annealed, and X-ray irradiated samples are shown as supplementary information online. The χ ranges used in Fourier transforms are 2 to 9 Å^−1^ and 3 to 11.5 Å^−1^ for Ce and Y EXAFS data, respectively. As shown in Supplementary Table S1 online, our Ce-L_3_-edge EXAFS curve-fitting revealed that the coordination number 5.6 ± 1.1 of the first (O) shell surrounding Ce in the X-ray irradiated sample is appreciably smaller than that in the as-grown sample (6.5 ± 1.3). This indicate that X-ray irradiation can indeed break Ce–O bonds, consistent with the Ce XANES results. The Y K-edge EXAFS results shown in Supplementary Table S1 online also show slightly decreased coordination number (from 6.9 ± 0.7 to 6.3 ± 0.8) of the first (O) shell surrounding Y as a result of X-ray irradiation, similar to the effect on the Ce–O bonds.

**Figure 5:**
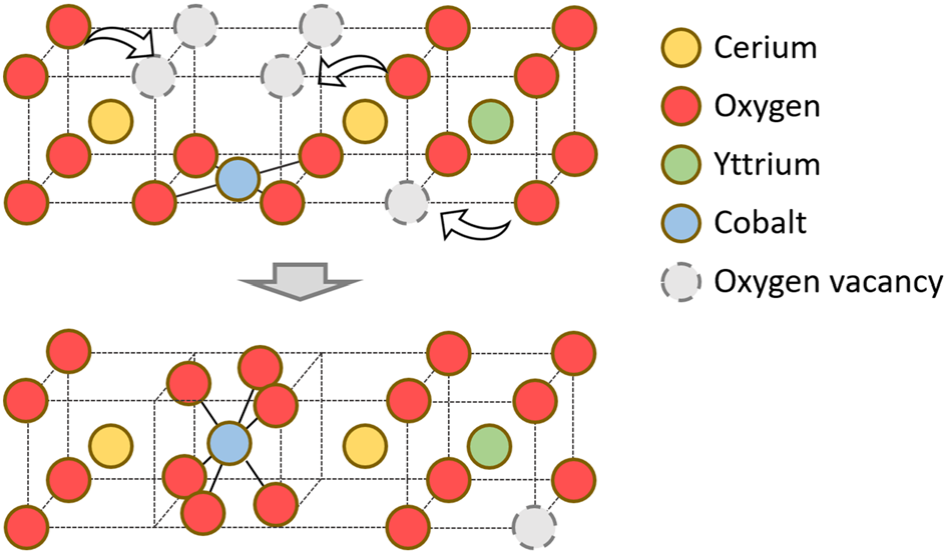
A simple model for the X-ray induced local structural change surrounding the Co dopant atoms.

As shown in Fig. 5, we propose the following model for the correlation between the breakaway of O atoms from Ce and the switching of Co-O4 structure to Co-O6 structure around Co atoms due to X-ray irradiation on the thermally annealed sample. Irradiating the sample with X-rays can break the Ce–O bonds, leading to diffuse of breakaway oxygen atoms through the thermally annealed (Y, Co)-codoped CeO_2_ sample, in which the Co dopant atoms are predominantly coordinated to 4 oxygen atoms in the square-planar geometry. As the breakaway oxygen atoms migrate, they either travel through oxygen-vacancy pathways created by the subvalent Y codopant or become trapped interstitial oxygen atoms in the sample. The migrating O may eventually leave the sample surface and largely increase the overall Ce^3+^ concentration in the host. However, some breakaway O atoms may jump into Co-O4 sites to form octahedral Co-O6 structure, leading to the observed reversal of thermally annealing effects.

## Conclusion

We have previously shown that Co in (Y, Co)-codoped nanocrystals of CeO_2_ can have two distinct types of oxygen coordination. After thermal annealing at elevated temperatures, the Co dopant atoms tend to form the energetically favored Co-O4 square-planar structure in the CeO_2_ host. Consequently, the band gap of the codoped CeO_2_ undergoes a dramatic reduction. In this work, we observed that X-ray irradiation can induce reversal of thermal annealing effects that additional Co–O bonds were formed and the Co dopant atoms were *pumped* back to the metastable octahedral Co-O6 environment as a result of X-ray irradiation. Ionizing X-ray radiation is normally known to break chemical bonds and cause radiation damage. The formation of Co–O bonds observed in this work appears to be a peculiar example of unconventional effects of X-rays on condensed matters. It also provides a special opportunity to fine tune and optimize the electronic properties of codoped CeO_2_ nanocrystals using thermal annealing and X-ray irradiation in opposing directions for technological applications.

## Methods

A polyol method was used to synthesize (Y, Co)-codoped CeO_2_ nanocrystal samples. Synchrotron-based XRD data was used to reveal the crystal structures of the samples and to determine particle sizes using the Scherrer equation. The Y and Co concentrations in the samples were obtained from inductively coupled plasma mass spectrometry (ICPMS) measurements. Thermally annealed samples were irradiated by synchrotron X-ray beam of spot size 1.5 mm × 0.5 mm and photon flux 1.2 × 10^12^ photons per second to investigate the effect of X-ray irradiation. To probe the local structural variation due to X-ray irradiation, Co K-edge and Ce L_3_-edge X-ray absorption near edge structure (XANES) and Co K-edge extended X-ray absorption fine structure (EXAFS) data were measured for the as-grown, annealed, and X-ray irradiated samples at beamline 07A of Taiwan Light Source (TLS) at National Synchrotron Radiation Research Center (NSRRC) in Taiwan.

## Supplementary Information

Supplementary Information.

## Data Availability

All data generated or analyzed during this study are included in this published article.
