# Supplementary material for: Formation of Co–O bonds and reversal of thermal annealing effects induced by X-ray irradiation in (Y, Co)-codoped CeO2 nanocrystals
Source: Sci Rep. 2022 Jan 28;12:1578. doi: 10.1038/s41598-022-05691-0 (PMC8799635; doi:10.1038/s41598-022-05691-0)
Supplement: Supplementary file 1 — Supplementary Information. [file 41598_2022_5691_MOESM1_ESM.docx]

**X-ray induced formation of Co-O bonds; Reversal of thermal annealing effects by x-ray irradiation in (Y, Co) codoped CeO_2_ nanocrystals**

Tai-Sing Wu ^1^, Sheng-Fu Chen^2^, Shih-Chang Weng^1^_,_ and Yun-Liang Soo*^1,2^

*^1^ National Synchrotron Radiation Research Center, Hsinchu, Taiwan*

*^2^Department of Physics, National Tsing Hua University, Hsinchu, Taiwan*

**Supplementary Information**

**FDMNES calculations**

The theoretical Co K-edge XANES spectra were calculated using Finite-Difference Method for Near Edge Structure (FDMNES) codes.^16^ The self-consistent mufﬁn-tin (MT) full-multiple-scattering (FMS) approach with the real Hedin-Lundqvist exchange-correlation potential was applied. Sparse solvers for the finite difference matrix were used in order to decrease the required computational time.^17^ Local structural information obtained from EXAFS analysis were used to construct the theoretical model. The calculated cluster radius is 2.1 Å that corresponds to 7 and 5 atoms for the octahedral model and the square planar model. For the comparison of experimental and simulated spectra, energy dependent broadening was then applied using the FDMNES implanted arctangent model to account for the broadening due both to the core level width and the final state width.

**Cerium L_3_-edge and Yttrium K-edge XAFS (XANES and EXAFS)**


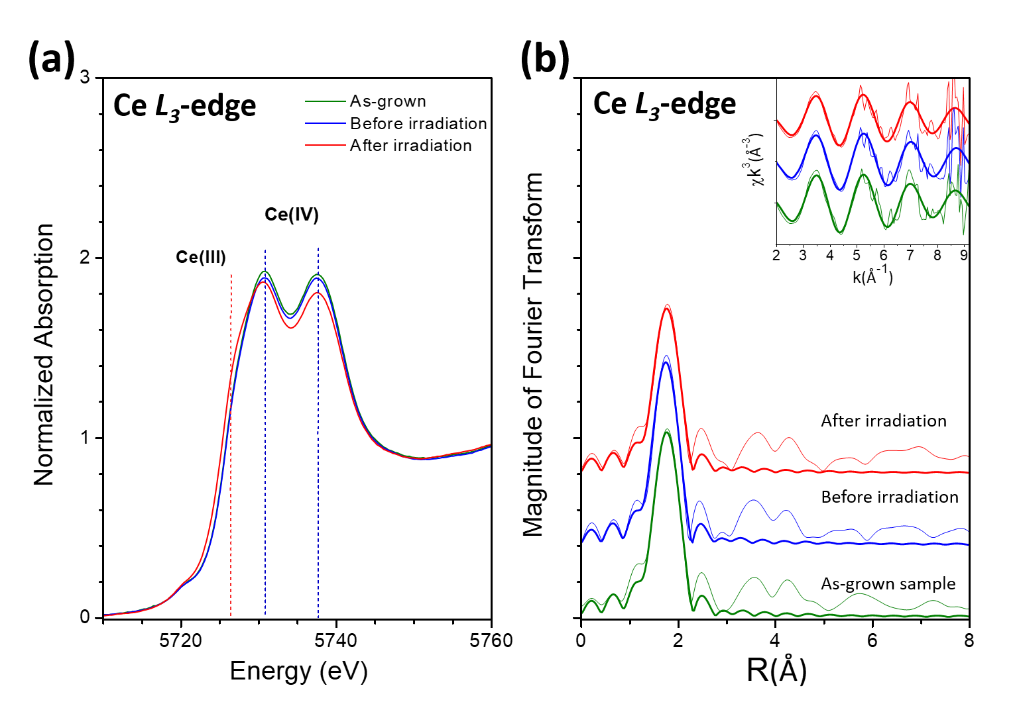


Supplementary Figure S1 – (a) Ce L_3_-edge XANES curves for (Y, Co)-codoped CeO_2_ samples. (b) Ce L_3_-edge EXAFS data for (Y, Co)-codoped CeO_2_ samples. Fine lines: experimental; Coarse lines: curve fitting. Curves have been shifted vertically for the sake of clarity.


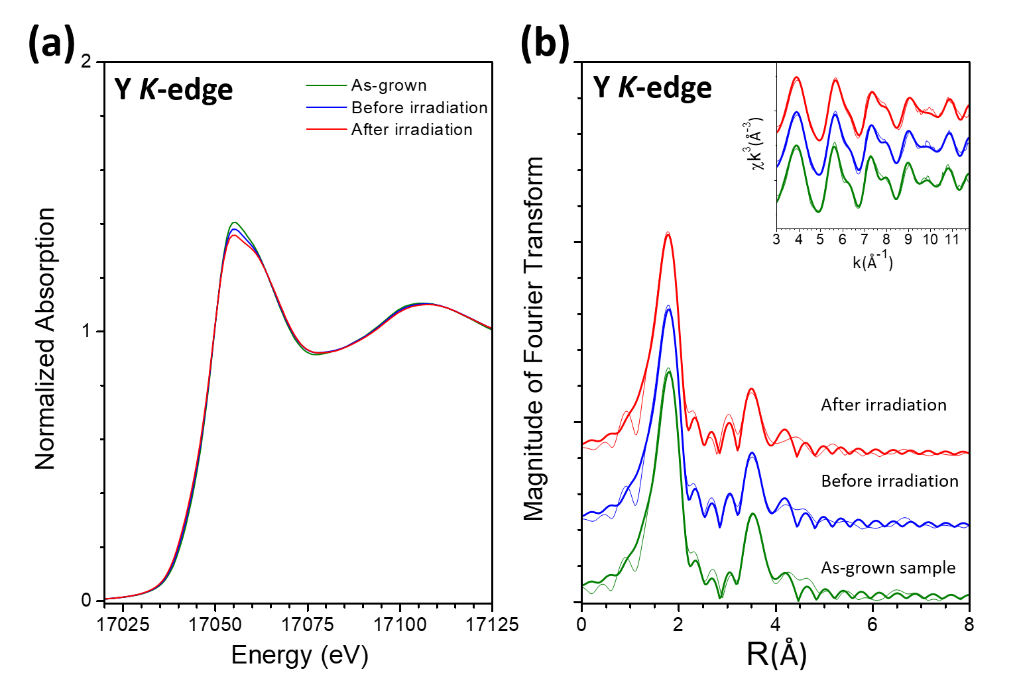


Supplementary Figure S2 – (a) Y K-edge XANES curves for (Y, Co)-codoped CeO_2_ samples. (b) Y K-edge EXAFS data for (Y, Co)-codoped CeO_2_ samples. Fine lines: experimental; Coarse lines: curve fitting. Curves have been shifted vertically for the sake of clarity.

**Supplementary Table S1.** Parameters of local structure around Ce atoms obtained from curve-fitting of the Ce L_3_-edge EXAFS. N is the coordination number. R is the bond length. σ^2^ is the Debye-Waller-like factor serving as a measure of local disorder. ΔE_0_ is the difference between the zero kinetic energy value of the sample and that of the theoretical model used in FEFF. R-factor is a residual factor representing the goodness of fit. Uncertainties were estimated by the double-minimum residue (2χ) method. The amplitude reduction factor ($S_{0}^{2}$) representing the central atom shakeup and shakeoff effects used in the curve-fitting is 0.73 as obtained from fitting the data of a bulk CeO_2_ model compound.

|  |  |  | ***R*** | **σ^2^** | **ΔE_0_** | **R-factor** |
| --- | --- | --- | --- | --- | --- | --- |
| **Sample** | **Bond** | ***N*** | **(Å)** | **(10^-3^Å^2^)** | **(eV)** | **(%)** |
| As-grown | Ce - O | 6.5 ± 1.3 | 2.27 ± 0.01 | 8.9 ± 3.1 | 4.3 ± 1.8 | 0.0101 |
| Before X-ray | Ce - O | 6.1 ± 1.0 | 2.26 ± 0.01 | 8.0 ± 2.3 | 3.0 ± 1.9 | 0.0082 |
| After X-ray | Ce - O | 5.6 ± 1.1 | 2.07 ± 0.01 | 8.2 ± 3.0 | 3.9 ± 2.1 | 0.0141 |

**Supplementary Table S2.** Parameters of local structure around Y atoms obtained from curve-fitting of the Y K-edge EXAFS. The amplitude reduction factor ($S_{0}^{2}$) used in the curve-fitting is 0.9 as obtained from fitting the data of a bulk Y_2_O_3_ model compound.

|  |  |  | ***R*** | **σ^2^** | **ΔE_0_** | **R-factor** |
| --- | --- | --- | --- | --- | --- | --- |
| **Sample** | **Bond** | ***N*** | **(Å)** | **(10^-3^Å^2^)** | **(eV)** | **(%)** |
| As-grown | Y - O | 6.9 ± 0.7 | 2.31 ± 0.01 | 10.4 ± 1.2 | -4.3 ± 1.2 | 0.0179 |
|  | Y - Ce | 2.3 ± 0.8 | 3.77 | 6.8 ± 2.1 | -7.5 ± 0.8 |  |
|  | Y - O | 5.8 ± 1.2 | 4.48 | 6.8 ± 2.1 | -6.4 ± 0.8 |  |
| Before X-ray | Y - O | 6.5 ± 0.6 | 2.29 ± 0.01 | 10.2 ± 1.1 | -4.6 ± 1.1 | 0.0137 |
|  | Y - Ce | 1.4 ± 0.5 | 3.77 | 4.7 ± 2.0 | -9.4 ± 2.4 |  |
|  | Y - O | 4.3 ± 0.9 | 4.48 | 4.7 ± 2.0 | -6.2 ± 0.8 |  |
| After X-ray | Y - O | 6.3 ± 0.8 | 2.29 ± 0.01 | 9.8 ± 1.6 | -4.9 ± 2.1 | 0.0888 |
|  | Y - Ce | 1.1 ± 0.6 | 3.77 | 4.3 ± 3.4 | -3.8 ± 1.7 |  |
|  | Y - O | 3.5 ± 1.2 | 4.48 | 4.3 ± 3.4 | -6.5 ± 1.7 |  |
